# The competitiveness analysis of shallot in Indonesia: A Policy Analysis Matrix

**DOI:** 10.1371/journal.pone.0256832

**Published:** 2021-09-03

**Authors:** Saptana, Endro Gunawan, Atika Dyah Perwita, Syahrul Ganda Sukmaya, Valeriana Darwis, Ening Ariningsih, Ashari

**Affiliations:** 1 Indonesian Center for Agricultural Socio Economic and Policy Studies, Bogor, Indonesia; 2 Faculty of Education and Teacher Training, Universitas Islam Negeri Walisongo Semarang, Semarang, Central Java, Indonesia; 3 Faculty of Agriculture, Universitas Jenderal Soedirman, Purwokerto, Central Java, Indonesia; Szechenyi Istvan University: Szechenyi Istvan Egyetem, HUNGARY

## Abstract

Shallot is a national strategic commodity in Indonesia, but it is development has a fundamental technical, socioeconomic, and policy support problems. Therefore, it is essential to know the competitiveness of shallot in Indonesia and the incentive policy to implement the comparative advantage to become a sustainable competitive advantage. The purposes of this study are to (1) analyze the profitability of shallot farming privately and socially, (2) analyze the competitiveness of shallot farming from a competitive and comparative advantage perspective, (3) review the impact of government policy on shallot farming, and (4) formulate incentive policies in the development of shallot commodities. The empirical results of the Policy Analysis Matrix revealed that shallot farming in production centers in Indonesia has both competitive and comparative advantages. The highest competitive and comparative advantages were found in the dry season in the upland of Malang district with the coefficient values of PCR (Private Cost Ratio) of 0.268–0.508 and DRCR (Domestic Resource Cost Ratio) of 0.208–0.323. The lowest competitive advantage was found in the lowland of East Lombok district in the dry season with a coefficient value of PCR 0.728–0.844. The lowest comparative advantage in the dry season was found in East Lombok district with a DRCR of 0.448, while in the rainy season, it was found in Wonosobo district with a DRCR of 0.522. These results mean that it is more profitable for Indonesia to increase domestic shallot production than to import. Improving shallot competitiveness can be carried out by implementing advanced technology, agricultural infrastructure, capacity building of farmers’ resources, and government incentive policies to increase productivity and competitiveness sustainability.

## 1. Introduction

The strengthening of trade liberalization provides new opportunities and new challenges that farmers and businesses must face in the supply chain of agricultural commodities. In terms of market demand, trade liberalization provides new opportunities due to an increasingly open market in line with eliminating various trade barriers between countries. Nevertheless, trade liberalization also poses severe problems if locally produced shallot cannot compete globally. A product can be competitive if it is of high quality, different from competing products, acceptable to consumers, and sustainable [1–3].

The previous studies revealed that the competitiveness is determined by the product value chain’s overall performance, both at the local and global levels [4, 5]. At the local level, it is determined by the ability to create added value through upgrading, strengthening local institutions, and the role of industry stakeholders. On the other hand, at the global level, the input-output structure of governance value chains, geographic scope, and management structure is influenced by the leadership of companies and industry organizations. In some agricultural products’ value chains, analysis and progress direction have increasingly oriented to the global market, so the value chain is often referred to as the global value chain (GVC) [6–8].

Each country has different policies in improving the competitiveness of its agricultural products. The implementation of policies in the agricultural sector carried out by countries in Asia and other developing countries are irrigation improvements, agricultural mechanization, land ownership status, and agricultural input subsidies [9–11]. The impact of implementing these policies has been enjoyed both by farmers and the country in terms of increased agricultural products’ competitiveness.

In the Regional Comprehensive Economic Partnership (RCEP) context, the added value could be maximally obtained if Indonesia participates in the regional and global value chains [12]. Opportunities for Indonesia’s agricultural products processing industry to take part in the regional and global value chains are still open [13–15]. Applying value chain analysis in horticultural subsectors can drive the country’s economic activities and provide a more equitable income distribution to businesses actors [16, 17]. Before a country enters the GVC, there are some indicators and criteria to review in advance for involvement in GVC to provide positive economic benefits. These criteria include vulnerability, treadmilling, leaps forward, and state institutional capacity [18]. The previous study revealed that transportation significantly influences the integrative supply chain and shallot products’ business performance [19].

The previous study using the trade specialty index (TSI) method revealed that plantation commodities are already at the maturation level of the world trade [20]. Meanwhile, food and horticultural commodities are at the introduction level or do not yet have comparative advantages. Multinational companies’ competitive strategy and product brands should focus on lowering costs, improving service techniques, and improving product quality and differentiation. Also, an industrial product’s design must be integrated into the entire process of goods product development [21, 22].

The characteristics of shallot farming in the production centers in Indonesia are (a) carried out on both the lowland wetland and upland dryland, (b) small scale and scattered, (c) low farmer capital capability, (c) non-optimal application of technology, both the use of seeds, cultivation and post-harvest, (d) excessive use of pesticides to control pests, (e) lack of use of certified seed/seedlings, (f) non-optimal development of shallot areas based on farmer corporations, and (g) inefficient product marketing system and high price fluctuations [23].

In analyzing the competitiveness of shallot farming, some questions that need to answer are as follows. (1) Does the comparative advantage of shallot farming in the market also have a competitive advantage? (2) How are the competitive and comparative advantages of lowland wetland and upland dryland agroecosystems, as well as in dry seasons and rainy seasons. (3) Does the current competitive position have sustainability prospects. (4) What are the incentive policy choices to realize the comparative advantage to become a sustainable competitive advantage?

Shallot is a national strategic commodity. However, its development face fundamental problems in technical, socioeconomic, and policy support. Based on these issues, this paper aimed to (1) analyze the profitability of shallot farming both privately and socially, (2) conduct shallot farming competitiveness analysis both competitive and comparative advantages, (3) review the impact of government policies on the shallot farming system, and (4) formulate incentive policies in the development of sustainable competitive shallot farming. This study provides some recommendations to improve the shallot competitiveness.

## 2. Literature review

Trade theory proposes that the concept of comparative advantage constructs a nation’s competitiveness. Formulated by Ricardo and Heckscher-Ohlin, comparative advantage assumed that trade flows are the consequence of differences in production costs among countries [24]. Comparative advantage is characterized by lower investment requirements to produce certain goods and higher productivity. It could be a trade-off between the comparative and competitive advantage, where increasing competitive advantage will counterweight by decreasing comparative advantage. There are three ways to increase competitive advantage, i.e., cost leader strategy, product differentiation, and focus on the niche market [25–27].

The determination of comparative advantage is generally approached by Revealed Comparative Advantage (RCA). It is accredited by the Ricardian comparative advantage concept and usually relates to an index, called the Balassa index, proposed by Balassa [28]. Comparative advantage is "feasible" if RCA>1. Some studies in Turkey, the EU, and Hawaii use this RCA as an indicator to determine which commodities have a comparative advantage [29–31]. Hawaii possessed an above-average comparative advantage in the following six products: anthuriums (with an RCA score of 16.12), macadamia nuts (6.48), cut orchids (6.26), pineapples (5.36), papayas (3.05), and raw sugar (2.62) [31].

In the Southeast Asia region (ASEAN) case, using Revealed Symmetric Comparative Advantage (RSCA) and Trade Balance Index (TBI) to seek comparative advantage in six countries in ASEAN, the results of the study confirmed that, on average, the comparative advantage of six countries increases [32]. Thailand and Vietnam’s comparative advantage and trade balance changes are more dynamic than the other four countries. The finding also revealed that there was competition and complementary amongst six countries. The previous study in ASEAN countries reveals that in confronting the challenge of competition in the era of the free market, the competitiveness of export of agricultural products still encounters hindrances in competing in the market [33]. Indonesia is a net-exporter and has a comparative advantage mostly from animal or vegetable fats and oil, Malaysia’s primarily are from rubber and articles thereof, Singapore and Thailand’s mainly from organic chemicals, and the Philippines’s mostly from electrical and electrical equipment [34]. Meanwhile, India has a comparative advantage in vegetable, fruits, and flower trade in the Asian, EU, and North American markets compared to selected other South East Asian countries [35]. Another study in Turkey and European Union states that European Union has more comparative advantage in agricultural items than Turkey [36].

Besides RCA, other indicators to determine competitive and comparative advantage are the Private Cost Ratio (PCR) and Domestic Resource Cost Ratio (DRCR). The two indicators are usually used when the Policy Analysis Matrix (PAM) is used as an analytical tool. The calculation is somewhat complex because it considers private and social costs, domestic components, and tradable inputs/outputs. The private cost ratio is the ratio between private domestic input costs and the difference between revenues and private tradable input costs [37]. Farming activity is competitive if the PCR value <1. Domestic Resource Cost Ratio (DRCR) is an indicator of comparative advantage that shows the number of domestic resources that can be saved to generate one foreign-exchange unit. Farming activities of a commodity are efficient in conditions without any government policy or have a comparative advantage if the DRCR value <1.

There are very few international studies examining the comparative advantage of shallots using PAM. The previous study in Ghana was carried out to determine the present status of the shallot farming enterprise, such as farmer characteristics, crop pattern, use of input, and the limitation of shallot farming [38]. The previous study in Thailand also discussed the impact of trade liberalization on shallot farming. The study result explains that the policies for coping with the impacts have not been effective, as reflected in increased product quality [25]. Study in Tanzania used DRC to measure the comparative advantage of rice, maize cotton, dan coffee [39].

As a producer and consumer of shallots, some studies have been conducted in association with the commodity in Indonesia. The topic of the study covers several aspects such as marketing and supply chain management [40–44], also on technical efficiency and sustainability of cultivation [45–50]. With the opening of international trade, several studies are interested in exploring the competitiveness of shallots in several production centers.

The previous study in Cirebon, Brebes, and Tegal districts shows that shallot farming in the three districts is financially profitable but economically unprofitable, so this farming system has no comparative advantage but has a competitive advantage [51]. The study in Ngajuk district, East Java province, shows relatively similar results [46]. The previous study in Samosir Regency, North Sumatra province and Pamekasan district, East Java shows that the shallot farming has competitive and comparative advantages [52, 53]. Thus, shallot is more advantageous to be produced domestically than imported from other countries [54, 55].

## 3. Methodology

### 3.1 Research framework

The concept of competitiveness was initially based on Adam Smith’s Theory of Absolute Advantage (1776), that suggests that welfare is a cluster of endowments [56, 57]. Ricardo’s Law of Comparative Advantage further refined Adam Smith’s Theory of Absolute Advantage. The Law states that even if a country does not have an absolute advantage in producing two types of commodities compared to other countries, mutually beneficial trade can still occur as long as there is a difference in the price ratio between countries when compared to no trade [56–58]. Meanwhile, Heckscher-Ohlin’s theory [56, 59], a theory of comparative advantage in international trade, states that “countries in which capital is relatively plentiful and labour relatively scarce will tend to export capital-intensive products and import labour-intensive products, while countries in which labor is relatively plentiful and capital relatively scarce will tend to export labor-intensive products and import capital-intensive products” [56, 60]. Competitiveness is defined as "the ability of a sector, industry or firm to compete successfully to achieve sustainable growth within the global environment while earning at least the opportunity cost of return on resources employed" [61].

We used Policy Matrix Analysis (PAM) tools [62, 63] to analyze the competitiveness and impact of government policies on shallot farming in the shallot production center area in Indonesia. This method has been widely implemented [55, 64–66].

### 3.2 Study area

The study was conducted in nine districts in six provinces, namely (a) Majalengka and Garut districts, West Java; (b) Brebes and Wonosobo districts, Central Java; (c) Nganjuk and Malang districts, East Java; (d) Solok district, West Sumatra; (e) Enrekang district, South Sulawesi; and (f) East Lombok district, West Nusa Tenggara. The study sites were selected purposively based on the following criteria: (1) a shallot production center in Indonesia, (2) different agroecosystems: wetland in the lowland area (wetland-lowland) and dryland in the upland area (dryland-upland), and (3) representing the area of Java island and outside Java island. Six districts represent the wetland-lowland production centers (Majalengka, Garut, Brebes, Nganjuk, Enrekang, and East Lombok) and three districts represent the dryland-upland production centers (Wonosobo, Malang, and Solok).

### 3.3 Sampling methods

This study employed *a multistage sampling procedure* as follows. First, purposive sampling was used to select the districts and provinces according to the criteria provided previously. Second, stratified sampling was employed to select farmers based on the land area and the technological innovation used by farmers. Third, simple random sampling was used to select 10–20 shallot farmers in each study site to participate in the focus group discussion (FGD), with a total of 110 farmers. The analysis was carried out on an average basis per production cycle per hectare according to two agroecosystems, i.e., wetland-lowland and dryland-upland, and two planting seasons, i.e., dry season and rainy season.

### 3.4 Methods of analysis

This study applied the Policy Analysis Matrix (PAM), where economic activities can be viewed from two perspectives: private and social perspectives [62–66]. Based on these perspectives. two main things should be explained: separation of cost components into tradable goods and domestic factors, and assessment of shadow (social) prices of inputs and outputs.

#### 3.4.1 Allocation of tradable input and domestic factor cost components

In PAM analysis, inputs used in the production process are separated into (a) tradable inputs (tradable goods) and (b) domestic factors (non-tradable goods). The first category inputs are the inputs that can be traded in the international market. In contrast, the second category inputs are the inputs that cannot be traded in the international market. In more detail, tradable goods are goods that are (1) exported or imported, (2) substitutes closely related to other types exported or imported, and (3) commodities other than the above and protected by the government, which actually can be traded internationally [62, 63, 67].

Two approaches can be used to allocate costs into tradable input and domestic factor components, namely the total approach and the direct approach. This study used a direct approach by assuming that the entire costs of tradable inputs, both imported and domestic production, are assessed as tradable input components. This approach is used because additional demand for tradable inputs, both imported and domestically produced, can be influenced by international trade [63].

The direct approach to vegetable commodities has been widely applied [64, 65]. In this study, shallot as the output is assumed to be 100 percent tradable goods. Meanwhile, inputs considered 100 percent tradable are shallot seeds, Urea fertilizer, SP-36, Potassium chloride (KCl), NPK, PPC, and pesticides. On the other hand, inputs assumed to be 100 percent domestic factors are organic fertilizer, labor, land rent value, and capital interests.

The allocation of tradable inputs and domestic factors for transportation activities was done based on interviews with various shallot business actors. Labor costs in the transportation process are domestic factors, and transport costs representing transportation equipment rental are components of tradable inputs. The distribution of tradable components and domestic factors on post-harvest handling costs was also based on direct interviews with shallot business actors. Material costs in the post-harvest handling process are tradable inputs, while labor costs are domestic factors. The results of cost allocation into tradable inputs and domestic factors are presented in the S1 File.

#### 3.4.2 Social pricing

This study set two levels of price for each input and output, namely, the actual prices in the market (private prices) and the shadow prices (social prices). Private price is the market price that farmers receive in selling their produce or the price paid in purchasing production factors. Shadow price occurs in the economy in a state of perfect competition and balance conditions [68]. Furthermore, shadow prices are generally determined by issuing distortions due to government policies such as subsidies, taxes, minimum wage determination, price policy, and other policies related to the shallot system. In this study, traded commodities were approached with border prices. Exported commodities used FOB (free on board) prices, while imported commodities used CIF (cost, insurance, freight) prices. Meanwhile, for domestic factors, the opportunity costs or the average prices in each location were used. The calculation mechanisms were as follows.

1. The shadow price of shallot seeds was based on the FOB price of USD 3,350/kg, then converted to rupiah, with a conversion rate of IDR 14,173/USD to become IDR 47,479/kg. The next step was to calculate it for each location by taking into account export taxes, value-added taxes, and transfer fees from the port to wholesalers to obtain the social price of shallot seeds.
2. The payment price for Urea fertilizer was based on its FOB price at USD 0.134/kg, then converted to rupiah with a conversion rate of IDR 14,173/USD to become IDR 1,899/kg. The next step was to calculate it for each location by taking into account export taxes, value-added taxes, and transfer fees from the port to wholesalers to obtain the social price of Urea fertilizer.
3. The shadow price of SP-36 fertilizer was based on the CIF price of USD 0.231/kg, then converted to rupiah with a conversion rate of IDR 14,173/USD to become IDR 3,274/kg. The next step was to calculate it for each location by taking into account import tariffs, value-added taxes, transfer fees from the port to wholesalers, and transfer fees from wholesalers to farmers to obtain the social price of SP-36 fertilizer.
4. The shadow price of NPK/Phonska fertilizer was based on the CIF price of USD 0.401/kg, then converted to rupiah with a conversion rate of IDR 14,173/USD to become IDR 5,683/kg. The next step was to calculate it for each location by taking into account import tariffs, value-added taxes, transfer fees from the port to wholesalers, and transfer fees from wholesalers to farmers to obtain the social price of NPK/Phonska fertilizer.
5. The shadow prices of organic/manure fertilizers, liquid-complementary fertilizer (PPC), and dolomite were approximated using the actual average prices in each study location due to the many types and various contents.
6. The shadow price of pesticide was approximated by the actual average price in each study location, minus the import tariff of 10% and the value-added tax of 10% to obtain the social price of pesticide.
7. The output price of shallot was based on the FOB price of USD 1,388/kg, then converted to rupiah with a conversion rate of IDR 14,173/USD to become IDR19,677/kg. The next step was to calculate it for each location by taking into account export taxes, value-added taxes, and transfer fees from the port to wholesalers to obtain the social price of shallot.
8. The output price of shallot was based on the FOB price of USD 1,388/kg, then converted to rupiah with a conversion rate of IDR 14,173/USD to become IDR 19,677/kg. The next step was to calculate it for each location by taking into account export taxes, value-added taxes, and transfer fees from the port to wholesalers to obtain the social price of shallot.
9. The shadow price of land was approximated by the value of the land rent in each study site. This was based on the fact that the land rental market mechanism was running well, as indicated by the running of land rent practices in the area.
10. The shadow price of the capital interest rate used the real interest rate, calculated by subtracting the actual interest rate from the inflation rate that occurred. Because most shallot farmers had access to BRI and BRI Units, the actual interest rate used the interest rate BRI’s KUPEDES was 3.31% per 4 months, with an inflation rate of 0.55% per month or 2.2% per 4 months so that the shadow price of capital interest was 1.31% per planting season (4 months).
11. The shadow price of the rupiah exchange rate against the dollar used the average actual exchange rate in 2019. This was based on the fact that Indonesia follows a floating exchange rate regime so that the shadow price of the rupiah exchange rate against the dollar was IDR 14,173/USD.

#### 3.4.3 Policy analysis matrix

The calculation of PAM consists of four steps: (1) determination of the complete physical inputs and outputs of the analyzed economic activity), (2) separation of all costs into components of tradable inputs and domestic factors, as well as calculating the revenues, (3) estimation of shadow prices of inputs and outputs, and (4) compilation and calculation of PAM analysis and various indicators resulted from PAM analysis. PAM preparation was done after all data at the farm and trade actor levels are obtained. PAM preparation was done by using an input-output structure at farm and trade actor levels. With this calculation, both private and social benefits can be obtained. The impacts of government policies applied to both inputs, outputs, and inputs and outputs altogether also can be known.

PAM analysis provides information about profitability, commodity competitiveness from economic efficiency (comparative advantage) and financial efficiency (competitive advantage), and the impacts of government policies on the commodity system. The PAM table used in this study [62, 63] is presented in Table 1, and the PAM results for shallot farming in Indonesia’s shallot production centers are presented in the S2 File.

**Table 1:** Policy Analysis Matrix (PAM).

	Revenues	Costs		Profit
		*Tradable inputs*	*Domestic factors*	
Private prices	A	B	C	D
Social prices	E	F	G	H
Divergences	I	J	K	L

Source: [62, 63]

Table notes

Private profits (PP): D = A–(B + C)

Social profits (SP): H = E–(F + G)

Output transfers (OT): I = A–E

Input transfers (IT): J = B–F

Factor transfers (FT): K = C–G

Net transfers (NT): L = D–H

Ratio indicators for comparison of unlike outputs

Private cost ratio (PCR) = C/(A-B)

Domestic resource cost ratio (DRCR) = G/(E-F)

Nominal protection coefficient on tradable outputs (NPCO) = A/E

Nominal protection coefficient on tradable inputs (NPCI) = B/F

Effective protection coefficient (EPC) = (A-B)/(E-F)

Profitability coefficient (PC) = D/H

Subsidy ratio to producers (SRP) = L/E.

## 4. Results and discussion

### 4.1 Social cost determination

The social price calculation in this study used adjustments as done by [68]. Shadow prices are generally determined by issuing distortions due to government policies such as subsidies, taxes, minimum wage determinations, pricing policies, and other policies. In this study, goods traded were approached with border prices. Exported goods used FOB price, and imported goods used the CIF price. Meanwhile, domestic factors were approached with the opportunity cost or the average price in each location. The social price for tradable goods can be seen in Table 2.

**Table 2:** Social price for tradable goods.

No	Components	Private price		Social price	
		USD/kg	IDR/kg	USD/kg	IDR/kg
1	Seed (FOB)	2.469	35,000	3.350	47,479
2	Urea (FOB)	0.127	1,800	0.314	1,899
3	SP-36 (CIF)	0,141	2,000	0.231	3,274
4	NPK (CIF)	0.162	2,300	0.401	5,683
5	KCl (CIF)	0.353	5,000	0.308	4,365
6	Shallot (FOB)	0.981	13,900	1.388	19,477

Note

• FOB (free on board), CIF (cost, insurance, freight)

• Exchange rate: 1 USD = IDR 1.4173

• The shadow price of pesticides was approached with the actual average price at each study site in Malang and Solok districts, then reduced by import tariff of 10% and value-added tax of 10%. Social prices are obtained from pesticides.

• The actual interest rate calculated by reducing the actual interest rate with the inflation rate. Because most of shallot farmers have access to BRI and BRI Unit, then the actual interest rate used the interest rate "Kupedes" BRI of 3.31% per 4 months, with an inflation rate of 0.55% per month or 2.2% per 4 months, so that the shadow price of capital interest was 1.31% per growing season (4 months).

### 4.2 Competitive and comparative advantages

The results of costs and financial (private) profit analysis show that shallot farming in shallot production centers, both in the wetland-lowland and dryland-upland of Indonesia, was profitable, both in the dry and rainy seasons. In general, social or economic profits were higher than financial or private profits, and the profits in the dry season were larger than those in the rainy season.

The financial profit (PP) of shallot farming was highest in Malang district in the rainy season (IDR 60,561,153/ha/season). Simultaneously, the lowest was found in Majalengka district in the rainy season (IDR 10,939,865/ha/season). The largest economic (SP) profit of shallot farming was found in Enrekang district in the dry season with a profit of IDR 186,838,618/ha/season, while in the lowest was in Majalengka district in the rainy seasson at IDR 88,764,893/ha/season. The results of this study are in line with the previous study [69], which concluded that the shallot farming in Solok district was profitable, providing a private profit of IDR 34,269,456/ha/season and a social profit of IDR 92,203,432/ha/season. The amount of costs and profits of farming privately and socially in each location is presented in Table 3.

**Table 3:** Values of PP, SP, PCR, and DRCR of shallot farming per ha per season in Indonesia’s shallot production centers, dry season 2019 and rainy season 2019/2020.

		PP (IDR)	SP (IDR)	PCR	DRCR
A	Wetland-lowland				
1	Majalengka				
	a. Dry season	36,987,612	114,127,508	0.633	0.357
	b. Rainy season	10,939,865	44,464,958	0.837	0.556
2	Garut				
	a. Dry season	55,157,060	132,933,801	0.459	0.259
	b. Rainy season	13,988,113	46,254,247	0.793	0.534
3	Brebes				
	a. Dry season	47,887,737	125,964,491	0.519	0.289
	b. Rainy season	11,723,699	44,798,652	0.820	0.542
4	Nganjuk				
	a. Dry season	48,052,540	95,903,027	0.538	0.366
	b. Rainy season	45,503,923	88,764,893	0.604	0.436
5	Enrekang				
	a. Dry season	38,669,209	186,838,618	0.605	0.239
	b. Rainy season	41,880,364	87,107,540	0.551	0.370
6	East Lombok				
	a. Dry season	41,363,932	184,299,935	0.591	0.243
	b. Rainy season	31,732,561	86,177,895	0.629	0.383
B	Dryland-upland				
1	Wonosobo				
	a. Dry season	51,682,337	130,515,065	0.493	0.276
	b. Rainy season	14,345,073	48,568,693	0.788	0.522
2	Malang				
	a. Dry season	51,891,153	100,027,296	0.416	0.269
	b. Rainy season	60,561,153	83,920,966	0.268	0.208
3	Solok				
	a. Dry season	39,830,587	104,915,738	0.542	0.309
	b. Rainy season	31,078,066	63,825,264	0.616	0.437

Source: Primary data, 2020 (processed)

Competitiveness analysis can be seen from two perspectives: the competitiveness of financial or private perspective, that is a competitive advantage, and social or economic competitiveness is a comparative advantage. Shallot farming in Indonesia’s production centers has competitiveness both from the perspective of competitive advantage shown by the value of PCR<1 coefficient and comparative advantage reflected in the value of DRCR<1 coefficient. The coefficient values of PCR <1 and DCR <1 indicate that shallot farming in production centers in Indonesia has both competitive and comparative advantages. This means that to produce a one-unit value-added output at private prices and social prices only require less than one-unit domestic resource costs. In general, comparative advantages are higher than competitive advantages. The competitiveness of shallot farming in the dry season was higher than in the rainy season.

The lowest PCR and DRCR values of shallot farming, which means that it had the highest competitive and comparative advantages, were found in Malang district in the rainy season. In this location, the PCR value was 0.268 and the DRCR value was 0.208. Meanwhile, the highest PCR and DRCR values was found in Majalengka district in the rainy season, 0.837 and 0.556, respectively. The analysis results for the value of the DRCR coefficient are in line with those performed by previous study [70] but with different PCR values. This study results agree with the previous study results by [69], although with a different magnitude that concludes that shallot farming in Solok district has a competitive advantage with a PCR value at 0.24 and comparative advantage with DRCR of 0.04. The magnitude of the coefficient values of PCR and DRCR of shallot farming according to agroecosystem and location is presented in Table 3.

### 4.3 Impact of divergences and government policies

The measures of the impact of divergences or government policies on shallot farming in the production centers in the PAM consist of output transfers (OT), input transfers (IT), factor transfers (FT), and net transfers (NT). The relative measure is shown based on the nominal protection coefficient on tradable outputs (NPCO), nominal protection coefficient on tradable inputs (NPCI), effective protection coefficient (EPC), profitability coefficient (PC), and subsidy ratio to producers (SRP).

#### 4.3.1 Impact of government policies on output. The value of OT and NPCO values of shallot farming in Indonesia’s shallot production center areas showed in Table 4

**Table 4:** Values of OT and NPCO of shallot farming per ha per season in Indonesia’s shallot production centers, dry season 2019 and rainy season 2019/2020.

		OT (IDR)	NPCO
A	Wetland-lowland		
1	Majalengka		
	a. Dry season	(84,804,000)	0.648
	b. Rainy season	(40,536,000)	0.747
2	Garut		
	a. Dry season	(85,224,000)	0.647
	b. Rainy season	(40,816,000)	0.746
3	Brebes		
	a. Dry season	(84,205,505)	0.646
	b. Rainy season	(40,118,775)	0.745
4	Nganjuk		
	a. Dry season	(86,004,000)	0.645
	b. Rainy season	(74,004,000)	0.694
5	Enrekang		
	a. Dry season	(163,232,000)	0.495
	b. Rainy season	(62,020,000)	0.693
6	East Lombok		
	a. Dry season	(160,432,000)	0.499
	b. Rainy season	(60,270,000)	0.699
B	Dryland-upland		
1	Wonosobo		
	a. Dry season	(86,004,000)	0.645
	b. Rainy season	(41,336,000)	0.744
2	Malang		
	a. Dry season	(73,104,000)	0.704
	b. Rainy season	(48,268,500)	0.777
3	Solok		
	a. Dry season	(77,847,000)	0.648
	b. Rainy season	(45,586,383)	0.747

Source: Primary data, 2020 (processed)

The policy impacts on the output or production of shallots in the production centers in Indonesia can be seen from the values of the OT and NPCO indicators. The government policies in the field of output can be in trade policy such as export tax, value-added tax (VAT), import tariff, subsidy policy, and supporting policies. Output transfer is the difference between revenues calculated on the financial (private) prices and calculated based on social prices. Nominal protection coefficient on tradable outputs (NPCO) is an indicator of output transfer calculated by dividing (ratio of) revenues calculated based on financial (private) prices with revenues calculated based on social prices.

Table 4 presents the OT and NPCO values of shallot farming in Indonesia’s shallot production center areas. In general, shallot farming, both in the agroecosystem of wetland-lowland and dryland-upland and in the dry and rainy seasons, showed negative OT values. The NPCO values for shallot farming in Indonesia’s shallot production center areas were <1. Negative OT and NPCO values <1 indicate that shallot farmers received lower prices than they should in a perfectly competitive market condition. In other words, on the output side, shallot farmers experience disincentive in producing shallots. Previous studies [71] supported the empirical study in the field that the shallot market structure in Indonesia is an oligopsony market indicated by many sellers and fewer buyers.

The highest NPCO coefficient value of shallot farming was found on the dryland -upland of Malang district in the rainy season (0.777). Meanwhile, the lowest NPCO value was found in the wetland-lowland of Enrekang district in the dry season (0.495). These results mean that farmers in Malang district received the smallest disincentive in terms of the shallot’s output, and vice versa in Enrekang district. Shallot farmers received a higher selling price of shallots in the rainy season than in the dry season. Table 4 presents the NPCO values of shallot farming according to agroecosystem and location.

#### 4.3.2 Impact of government policy in tradable inputs

The impact of government policy in tradable inputs on shallot farming is indicated by the values of input transfers (IT) and the nominal protection coefficient on tradable inputs (NPCI). Meanwhile, the impact of divergence or government policy in domestic factors is indicated by the factor transfers (FT).

Government policies in tradable inputs and domestic factors can be in the form of trade policy (import tariffs, quotas/import restrictions), input subsidies, taxes (value-added tax/VAT), while other divergences may be due to market distortions. Input transfers show the difference between the costs of tradable inputs at private prices and the costs of tradable inputs at social prices. The nominal protection coefficient on tradable inputs (NPCI) is an input transfer indicator which is a ratio between tradable input costs calculated at private prices and tradable input costs calculated at social prices. Information on the values of IT, NPCI, and FT of shallot farming on wetland-lowland and dryland-upland agroecosystems in Indonesia is presented in Table 5.

**Table 5:** Values of IT, NPCI, and FT of shallot farming per ha per season in Indonesia’s shallot production centers, dry season 2019 and rainy season 2019/2020.

		IT (IDR)	NPCI	FT (IDR)
A	Wetland-lowland			
1	Majalengka			
	a. Dry season	(8,032,530)	0.873	368,426
	b. Rainy season	(7,311,705)	0.879	300,798
2	Garut			
	a. Dry season	(7,679,955)	0.876	232,695
	b. Rainy season	(9,217,955)	0.850	668,089
3	Brebes			
	a. Dry season	(6,520,505)	0.892	391,753
	b. Rainy season	(7,403,705)	0.876	359,882
4	Nganjuk			
	a. Dry season	(38,779,931)	0.573	626,417
	b. Rainy season	(31,543,705)	0.628	800,675
5	Enrekang			
	a. Dry season	(15,648,331)	0.799	585,740
	b. Rainy season	(16,991,370)	0.733	198,546
6	East Lombok			
	a. Dry season	(18,056,000)	0.765	560,003
	b. Rainy season	(6,279,750)	0.896	455,084
B	Dryland-upland			
1	Wonosobo			
	a. Dry season	(7,799,955)	0.874	628,682
	b. Rainy season	(7,471,329)	0.875	358,949
2	Malang			
	a. Dry season	(25,131,454)	0.772	163,597
	b. Rainy season	(25,131,454)	0.772	222,767
3	Solok			
	a. Dry season	(13,037,200)	0.811	275,351
	b. Rainy season	(13,115,400)	0.804	276,215

Source: Primary data, 2020 (processed)

Input transfer indicators for shallot farming provided negative IT values, both on wetland-lowland and dryland-upland. Similarly, the values of NPCI obtained were <1 in all study sites. Negatively marked IT values and NPCI <1 indicate that farmers get incentives on the tradable input production side because they pay for production inputs lower than in perfectly competitive market conditions. The highest NPCI value in shallot farming was found in East Lombok district in the rainy season (0.896), while the lowest NPCI value was found in Nganjuk district in the dry season (0.573). These results mean that, in general, there is a policy in tradable inputs that benefits shallot farmers because farmers pay for tradable inputs lower than they should in a perfectly competitive market. Considering the government’s policies in tradable inputs in recent years, it turns out that the source of distortion is tariffs and value-added taxes. While subsidies have been partially abolished, farmers still receive subsidized fertilizer in some study sites [72].

Factor transfers for shallot generally showed positive values. The highest FT value was found in Nganjuk district in the rainy season (IDR 800,675/ha/season), while the lowest FT value was found in Malang district in the dry season (IDR 163,597/ha/season). This means there is a government policy or market distortion in domestic factors that harms farmers in producing shallot, causing farmers to pay for domestic factors slightly higher than they should. The primary source of price differences for the cost of domestic factors is sourced at capital interest. Although farmers paid more than the social price, the difference was relatively small [2].

#### 4.3.3 Impact of government policies on inputs-output

The impact of divergences and government policies on overall inputs and output in the shallot production area in Indonesia was demonstrated by the values of net transfer (NT), effective protection coefficient (EPC), profitability coefficient (PC), and subsidy ratio to producers (SRP). The analysis of the impact of government policy on inputs and outputs of shallot farming in shallot production centers in Indonesia is presented in Table 6.

**Table 6:** Values of NT, PC, EPC and SRP of shallot farming per ha per season in Indonesia’s shallot production centers, dry season 2019 and rainy season 2019/2020.

		PC	NT (IDR)	EPC	SRP
A	Wetland-lowland				
1	Majalengka				
	a. Dry season	0.568	(77,139,896)	(1.479)	(0.320)
	b. Rainy season	0.668	(33,525,093)	(1.326)	(0.210)
2	Garut				
	a. Dry season	0.568	(77,776,740)	(1.709)	(0.322)
	b. Rainy season	0.682	(32,266,134)	(1.434)	(0.201)
3	Brebes				
	a. Dry season	0.562	(78,076,753)	(1.613)	(0.328)
	b. Rainy season	0.666	(33,074,952)	(1.354)	(0.210)
4	Nganjuk				
	a. Dry season	0.688	(47,850,487)	(2.004)	(0.198)
	b. Rainy season	0.730	(43,260,970)	(2.05)	(0.179)
5	Enrekang				
	a. Dry season	0.399	(148,169,409)	(1.261)	(0.458)
	b. Rainy season	0.675	(45,227,176)	(1.926)	(0.224)
6	East Lombok				
	a. Dry season	0.415	(142,936,003)	(1.289)	(0.446)
	b. Rainy season	0.613	(54,445,334)	(1.583)	(0.272)
B	Dryland-upland				
1	Wonosobo				
	a. Dry season	0.566	(78,832,7270)	(1.656)	(0.326)
	b. Rainy season	0.667	(34,223,620)	(1.419)	(0.212)
2	Malang				
	a. Dry season	0.649	(48,136,143)	(2.078)	(0.195)
	b. Rainy season	0.782	(23,359,813)	(3.593)	(0.108)
3	Solok				
	a. Dry season	0.573	(65,085,151)	(1.612)	(0.295)
	b. Rainy season	0.713	(32,747,198)	(1.949)	(0.182)

Source: Primary data, 2020 (processed)

All the PC values for shallot farming in the production centers were positive (>0) but less than 1 in both agroecosystems and both dry and rainy seasons. The highest PC value was found on the dryland-upland of Malang district in the rainy season (0.782), while the lowest PC value was on the wetland-lowland of Enrekang district in the dry season (0.399). Government policies or market distortions that occur in shallot farming serve as a disincentive to shallot farmers. This means that farmers get a smaller profit than they should if the market mechanisms compete perfectly.

The NT values of shallot farming in the shallot production centers in both agroecosystems in Indonesia and both dry and rainy seasons were marked negative. The largest negative NT value was found on the wetland-lowland of Enrekang district in the dry season (–IDR 148,169,409/ha/season), while the smallest negative NT value was on the dryland-upland of Malang district in the rainy season (–IDR 23,359,813). Government policies or market distortions that occurred in shallot farming inputs (tradable inputs and domestic factors) and output served as a disincentive to shallot farmers as a whole. This means that farmers provide a relatively large transfer to the consumer community.

The EPC values of shallot farming in all study sites were negatively marked with a magnitude of <-1. The highest negative marked EPC value was found on the dryland-upland of Malang district in the rainy season of -3,593, while the lowest negative marked EPC value was on the wetland-lowland of Enrekang district in the dry season with a value of -1.261. Government policies or market distortions that occurred in shallot farming as a whole are a disincentive to shallot farmers. This means that farmers did not get adequate protection from existing policies or market distortions compared to when they are in the perfect market competition mechanisms.

The SRP values of shallot farming in both wetland-lowland and dryland-upland agroecosystems in all study sites were negative in both dry and rainy seasons. The largest negatively marked SRP value was found on the wetland-lowland agroecosystems of Enrekang district in the dry season (-0.458). Meanwhile, the lowest SRP value of shallot farming marked negatively was on the dryland-upland of Malang district in the rainy season (-0.108). This means that in general, government policies or market distortions applied on shallot farming have a detrimental impact on shallot farmers, both in the dry season and rainy season, because shallot farmers receive negative subsidies.

## 5. Conclusions

Shallot farming is financially (private) and economically (social) profitable. The highest financial profit was found on the dryland-upland of Malang district (IDR 60.56 million/planting season), while the lowest was on the wetland-lowland of Majalengka district (IDR 10.94 million/planting season). Meanwhile, the highest economic profit was found on the wetland-lowland of Enrekang (IDR 186.84 million/planting season), while the lowest was on the wetland-lowland of Majalengka district (IDR 44.46 million/planting season). In general, the financial profits received by farmers were smaller than the economic profits. This shows that shallot farmers in Indonesia experienced a disincentive in producing shallots because they received lower profits than they should. Incentive policies are needed to make farmers continue to plant shallots by encouraging market mechanisms to run perfectly, especially during the harvest season.

The study results show that shallot farming in production centers in Indonesia has a competitive advantage with a PCR coefficient value <1 and a comparative advantage as indicated by a DRCR coefficient value <1. The highest competitive advantage was found in the dry season on the wetland-lowland in Garut district with a PCR value of 0.459, while the lowest was on the wetland-lowland of Majalengka district in the rainy season with a PCR value of 0.837. Meanwhile, the highest comparative advantage was found in the wetland-lowland of Enrekang district in the dry season with a DRCR of 0.239, while the lowest was in the wetland-lowland of Majalengka district in the dry season with a DRCR value of 0.556. This means that to produce one unit of added value at private prices and social prices, the use of domestic resources is less than one unit. For Indonesia, in terms of efficiency in domestic resources, it is more profitable to increase domestic shallot production than to import.

The values of tradable inputs in all study locations were negative. This shows that the impact of government policies (market distortion) on tradable inputs was a disincentive because farmers have to pay for tradable inputs higher than they should. This phenomenon occurs mainly in the inputs of shallot seeds, non-subsidized fertilizers, and pesticides. The output transfer values in all research locations were negative, reflecting that farmers accepted the selling price of shallots lower than it should be. Meanwhile, on domestic factors, farmers obtained incentives, although relatively limited. Overall, government policies or market distortions in the input and output sectors are detrimental to shallot farmers. The government’s policy that is deemed relevant is to reduce distortions caused by both market distortions and policy distortions by developing an efficient logistics system and smooth market information services.

This study suggests that the government develop shallot farming in production centers with an agricultural area approach to achieve an efficient business scale. Therefore, it is necessary to support agricultural infrastructure, the use of certified superior shallot seeds, complete and balanced fertilization, organic fertilizers, agricultural mechanization for soil processing with rotary hand tractors, and provision of storage warehouses and dryers. Policies to increase the availability of certified superior seeds and fertilizers at the right time and location and stabilize the price of shallots at the main harvest time are also needed.

## Supporting information

S1 File (DOCX)

S2 File (DOCX)
