# Supplementary material for: The competitiveness analysis of shallot in Indonesia: A Policy Analysis Matrix
Source: PLoS One. 2021 Sep 3;16(9):e0256832. doi: 10.1371/journal.pone.0256832 (PMC8415590; doi:10.1371/journal.pone.0256832)
Supplement: S1 File — (DOCX) [file pone.0256832.s001.docx]

S1 File

S1. Allocation of shallot production costs into tradable input and domestic factor components in shallot production center area of Indonesia, 2019-2019/2020

|  |  | Tradable inputs (%) | Domestic factors (%) |
| --- | --- | --- | --- |
| A | Production inputs |  |  |
| 1 | Shallot seed | 100 | 0 |
| 2 | Organic fertilizer | 0 | 100 |
| 3 | Anorganic fertilizer |  |  |
|  | a. Urea/ZA | 100 | 0 |
|  | b. SP-36 | 100 | 0 |
|  | c. NPK/PONSKA | 100 | 0 |
|  | d. KCl | 100 | 0 |
|  | e. PPC | 100 | 0 |
| 4 | Dolomite | 0 | 100 |
| 5 | Pesticides | 100 | 0 |
| B | Labor |  |  |
| 1 | Seed preparation | 0 | 100 |
| 2 | Land processing | 0 | 100 |
| 3 | Planting | 0 | 100 |
| 4 | Fertilizer application | 0 | 100 |
| 5 | Maintainance | 0 | 100 |
| 6 | Harvest and transportation | 33.65 | 66.35 |
| 7 | Post-harvest | 28.48 | 71.52 |
| C | Irrigation costs |  |  |
| 1 | Fuel | 100 | 0 |
| 2 | Pump machine rent | 67 | 33 |
| D | Land rent | 0 | 100 |
| E | Interest rate | 0 | 100 |
