# Supplementary material for: The competitiveness analysis of shallot in Indonesia: A Policy Analysis Matrix
Source: PLoS One. 2021 Sep 3;16(9):e0256832. doi: 10.1371/journal.pone.0256832 (PMC8415590; doi:10.1371/journal.pone.0256832)
Supplement: S2 File — (DOCX) [file pone.0256832.s002.docx]

S2 File:

S2a. Policy Analisys Matrix of shallot farming on the wetland-lowland of Majalengka district in dry season 2019.

|  | Revenues  (IDR) | Costs | | Profits  (IDR) |
| --- | --- | --- | --- | --- |
|  |  | Tradable inputs (IDR) | Domestic factors (IDR) |  |
| Private prices | 156,000,000 | 55,234,220 | 63,778,168 | 36,987,612 |
| Social prices | 240,804,000 | 63,266,750 | 63,409,742 | 114,127,508 |
| Divergences | (84,804,000) | (8,032,530) | 368,426 | (77,139,896) |

S2b. Policy Analisys Matrix of shallot farming on the wetland-lowland of Majalengka district in rainy season 2019/2020.

|  | Revenues  (IDR) | Costs | | Profits  (IDR) |
| --- | --- | --- | --- | --- |
|  |  | Tradable inputs (IDR) | Domestic factors (IDR) |  |
| Private prices | 120,000,000 | 53,075,104 | 55,985,031 | 10,939,865 |
| Social prices | 160,536,000 | 60,386,809 | 55,684,233 | 44,464,958 |
| Divergences | (40,536,000) | (7,311,705) | 300,798 | (33,525,093) |

S2c. Policy Analisys Matrix of shallot farming on the wetland-lowland of Garut district in dry season 2019.

|  | Revenues  (IDR) | Costs | | Profits  (IDR) |
| --- | --- | --- | --- | --- |
|  |  | Tradable inputs (IDR) | Domestic factors (IDR) |  |
| Private prices | 156,000,000 | 54,090,120 | 46,752,820 | 55,157,060 |
| Social prices | 241,224,000 | 61,770,075 | 46,520,124 | 132,933,801 |
| Divergences | (85,224,000) | (7,679,955) | 232,695 | (77,776,740) |

S2d. Policy Analisys Matrix of shallot farming on the wetland-lowland of Garut district in rainy season 2019/2020.

|  | | Revenues  (IDR) | Costs | | Profits  (IDR) |
| --- | --- | --- | --- | --- | --- |
|  |  |  | Tradable inputs (IDR) | Domestic factors (IDR) |  |
| Private prices | 120,000,000 | 52,267,504 | 53,744,383 | 13,988,113 |  |
| Social prices | 160,816,000 | 61,485,459 | 53,076,294 | 46,254,247 |  |
| Divergences | (40,816,000) | (9,217,955) | 668,089 | (32,266,134) |  |

S2e. Policy Analisys Matrix of shallot farming on the wetland-lowland of Brebes district in dry season 2019.

|  | Revenues  (IDR) | Costs | | Profits  (IDR) |
| --- | --- | --- | --- | --- |
|  |  | Tradable inputs (IDR) | Domestic factors (IDR) |  |
| Private prices | 153,595,000 | 54,070,320 | 51,636,943 | 47,887,737 |
| Social prices | 237800505 | 60590825 | 51,245,189 | 125,964,491 |
| Divergences | (84,205,505) | (6,520,505) | 391,753 | (78,076,753) |

S2f. Policy Analisys Matrix of shallot farming on the wetland-lowland of Brebes district in rainy season 2019/2020.

|  | | Revenues  (IDR) | Costs | | Profits  (IDR) |
| --- | --- | --- | --- | --- | --- |
|  |  |  | Tradable inputs (IDR) | Domestic factors (IDR) |  |
| Private prices | 117,375,000 | 52,267,504 | 53,383,797 | 11,723,699 |  |
| Social prices | 157493775 | 59671209 | 53023914.3 | 44798651.74 |  |
| Divergences | (40,118,775) | (7,403,705) | 359,882 | (33,074,952) |  |

S2g. Policy Analisys Matrix of shallot farming on the wetland-lowland of Nganjuk district in dry season 2019.

|  | | Revenues  (IDR) | Costs | | Profits  (IDR) |
| --- | --- | --- | --- | --- | --- |
|  |  |  | Tradable inputs (IDR) | Domestic factors (IDR) |  |
| Private prices | 156,000,000 | 51,985,310 | 55,962,150 | 48,052,540 |  |
| Social prices | 242,004,000 | 90,765,241 | 55,335,733 | 95,903,027 |  |
| Divergences | (86,004,000) | (38,779,931) | 626,417 | (47,850,487) |  |

S2h. Policy Analisys Matrix of shallot farming on the wetland-lowland of Nganjuk district in rainy season 2019/2020.

|  | Revenues  (IDR) | Costs | | Profits  (IDR) |
| --- | --- | --- | --- | --- |
|  |  | Tradable inputs (IDR) | Domestic factors (IDR) |  |
| Private prices | 168,000,000 | 53,163,280 | 69,332,797 | 45,503,923 |
| Social prices | 242,004,000 | 84,706,985 | 68,532,122 | 88,764,893 |
| Divergences | (74,004,000) | (31,543,705) | 800,675 | (43,260,970) |

S2i. Policy Analisys Matrix of shallot farming on the wetland-lowland of Enrekang district in dry season 2019.

|  | Revenues  (IDR) | Costs | | Profits  (IDR) |
| --- | --- | --- | --- | --- |
|  |  | Tradable inputs (IDR) | Domestic factors (IDR) |  |
| Private prices | 160,000,000 | 62,051,520 | 59,279,271 | 38,669,209 |
| Social prices | 323,232,000 | 77,699,851 | 58,693,531 | 186,838,618 |
| Divergences | (163,232,000) | (15,648,331) | 585,740 | (148,169,409) |

S2j. Policy Analisys Matrix of shallot farming on the wetland-lowland of Enrekang district in rainy season 2019/2020.

|  | Revenues  (IDR) | Costs | | Profits  (IDR) |
| --- | --- | --- | --- | --- |
|  |  | Tradable inputs (IDR) | Domestic factors (IDR) |  |
| Private prices | 140,000,000 | 46,687,320 | 51,432,316 | 41,880,364 |
| Social prices | 202,020,000 | 63,678,690 | 51,233,770 | 87,107,540 |
| Divergences | (62,020,000) | (16,991,370) | 198,546 | (45,227,176) |

S2k. Policy Analisys Matrix of shallot farming on the wetland-lowland of East Lombok district in dry season 2019.

|  | Revenues  (IDR) | Costs | | Profits  (IDR) |
| --- | --- | --- | --- | --- |
|  |  | Tradable inputs (IDR) | Domestic factors (IDR) |  |
| Private prices | 160,000,000 | 58,823,880 | 59,812,188 | 41,363,932 |
| Social prices | 320,432,000 | 76,879,880 | 59,252,185 | 184,299,935 |
| Divergences | (160,432,000) | (18,056,000) | 560,003 | (142,936,003) |

S2l. Policy Analisys Matrix of shallot farming on the wetland-lowland of East Lombok district in rainy season 2019/2020.

|  | Revenues  (IDR) | Costs | | Profits  (IDR) |
| --- | --- | --- | --- | --- |
|  |  | Tradable inputs (IDR) | Domestic factors (IDR) |  |
| Private prices | 140,000,000 | 54,366,320 | 53,901,119 | 31,732,561 |
| Social prices | 200,270,000 | 60,646,070 | 53,446,035 | 86,177,895 |
| Divergences | (60,270,000) | (6,279,750) | 455,084 | (54,445,334) |

S 2m. Policy Analisys Matrix of shallot farming on the dryland-upland of Wonosobo district in dry season 2019.

|  | Revenues  (IDR) | Costs | | Profits  (IDR) |
| --- | --- | --- | --- | --- |
|  |  | Tradable inputs (IDR) | Domestic factors (IDR) |  |
| Private prices | 156,000,000 | 54,050,120 | 50,267,543 | 51,682,337 |
| Social prices | 242,004,000 | 61,850,075 | 49,638,860 | 130,515,065 |
| Divergences | (86,004,000) | (7,799,955) | 628,682 | (78,832,727) |

S2n. Policy Analisys Matrix of shallot farming on the dryland-upland of Wonosobo district in rainy season 2019/2020.

|  | Revenues  (IDR) | Costs | | Profits  (IDR) |
| --- | --- | --- | --- | --- |
|  |  | Tradable inputs (IDR) | Domestic factors (IDR) |  |
| Private prices | 120,000,000 | 52,271,130 | 53,383,797 | 14,345,073 |
| Social prices | 161,336,000 | 59,742,459 | 53,024,848 | 48,568,693 |
| Divergences | (41,336,000) | (7,471,329) | 358,949 | (34,223,620) |

S 2o. Policy Analisys Matrix of shallot farming on the dryland-upland of Malang district in dry season 2019.

|  | Revenues  (IDR) | Costs | | Profits  (IDR) |
| --- | --- | --- | --- | --- |
|  |  | Tradable inputs (IDR) | Domestic factors (IDR) |  |
| Private prices | 173,820,000 | 85,013,776 | 36,915,071 | 51,891,153 |
| Social prices | 246,924,000 | 110,145,230 | 36,751,474 | 100,027,296 |
| Divergences | (73,104,000) | (25,131,454) | 163,597 | (48,136,143) |

S2p. Policy Analisys Matrix of shallot farming on the dryland-upland of Malang district in rainy season 2019/2020.

|  | Revenues  (IDR) | Costs | | Profits  (IDR) |
| --- | --- | --- | --- | --- |
|  |  | Tradable inputs (IDR) | Domestic factors (IDR) |  |
| Private prices | 167,790,000 | 85,013,776 | 22,215,071 | 60,561,153 |
| Social prices | 216,058,500 | 110,145,230 | 21,992,304 | 83,920,966 |
| Divergences | (48,268,500) | (25,131,454) | 222,767 | (23,359,813) |

S2q. Policy Analisys Matrix of shallot farming on the dryland-upland of Solok district in dry season 2019.

|  | Revenues  (IDR) | Costs | | Profits  (IDR) |
| --- | --- | --- | --- | --- |
|  |  | Tradable inputs (IDR) | Domestic factors (IDR) |  |
| Private prices | 143,000,000 | 55,992,770 | 47176643 | 39,830,587 |
| Social prices | 220,847,000 | 69,029,970 | 46901292 | 104,915,738 |
| Divergences | (77,847,000) | (13,037,200) | 275,351 | (65,085,151) |

S2r. Policy Analysis Matrix of shallot farming on the dryland-upland of Solok district in rainy season 2019/2020.

|  | Revenues  (IDR) | Costs | | Profits  (IDR) |
| --- | --- | --- | --- | --- |
|  |  | Tradable inputs (IDR) | Domestic factors (IDR) |  |
| Private prices | 134,685,000 | 53,822,770 | 49784164 | 31,078,066 |
| Social prices | 180,271,383 | 66,938,170 | 49507949 | 63,825,264 |
| Divergences | (45,586,383) | (13,115,400) | 276,215 | (32,747,198) |
